# Pharmacokinetic and pharmacogenetic determinants of the activity and toxicity of irinotecan in metastatic colorectal cancer patients

**DOI:** 10.1038/sj.bjc.6604673

**Published:** 2008-09-16

**Authors:** E Rouits, V Charasson, A Pétain, M Boisdron-Celle, J-P Delord, M Fonck, A Laurand, A-L Poirier, A Morel, E Chatelut, J Robert, E Gamelin

**Affiliations:** 1 Laboratoire d'Oncopharmacologie, Centre Paul-Papin, 2 rue Moll, Angers 49000, France; 2 Département de Pharmacologie INSERM U916, Institut Bergonié, 229 cours de l'Argonne, Bordeaux 33076, France; 3 Équipe d'accueil 3035, Université de Toulouse, Institut Claudius-Regaud, 20 rue du Pont Saint-Pierre, Toulouse 31052, France

**Keywords:** colorectal cancer, irinotecan, pharmacokinetics, UGT1A1 gene polymorphism, CYP3A4

## Abstract

This study aims at establishing relationships between genetic and non-genetic factors of variation of the pharmacokinetics of irinotecan and its metabolites; and also at establishing relationships between the pharmacokinetic or metabolic parameters and the efficacy and toxicity of irinotecan. We included 49 patients treated for metastatic colorectal cancer with a combination of 5-fluorouracil and irinotecan; a polymorphism in the UGT1A1 gene (TA repeat in the TATA box) and one in the CES2 gene promoter (830C>G) were studied as potential markers for SN-38 glucuronidation and irinotecan activation, respectively; and the potential activity of CYP3A4 was estimated from cortisol biotransformation into 6*β*-hydroxycortisol. No pharmacokinetic parameter was directly predictive of clinical outcome or toxicity. The AUCs of three important metabolites of irinotecan, SN-38, SN-38 glucuronide and APC, were tentatively correlated with patients' pretreatment biological parameters related to drug metabolism (plasma creatinine, bilirubin and liver enzymes, and blood leukocytes). SN-38 AUC was significantly correlated with blood leukocytes number and SN-38G AUC was significantly correlated with plasma creatinine, whereas APC AUC was significantly correlated with plasma liver enzymes. The relative extent of irinotecan activation was inversely correlated with SN-38 glucuronidation. The TATA box polymorphism of UGT1A1 was significantly associated with plasma bilirubin levels and behaved as a significant predictor for neutropoenia. The level of cortisol 6*β*-hydroxylation predicted for the occurrence of diarrhoea. All these observations may improve the routine use of irinotecan in colorectal cancer patients. UGT1A1 genotyping plus cortisol 6*β*-hydroxylation determination could help to determine the optimal dose of irinotecan.

Colorectal adenocarcinoma is a leading cause of cancer in developed countries and is responsible for 16 000 deaths every year in France (Hill and Doyon, 2005) and 200 000 in Europe (Boyle and Ferlay, 2005). This is a curable disease as long as metastatic dissemination has not occurred, and even in that case, the combination of chemotherapy and surgical removing of hepatic metastases is sometimes able to cure the patient (Bentrem *et al*, 2005). Colorectal cancer was considered 20 years ago as a globally chemoresistant disease and the only available drug was 5-fluorouracil (5-FU), which, however, provided low response rates and limited survival enhancement. Progress came first from a better understanding of 5-FU pharmacology: the combination with folinic acid and the prolongation of drug infusion have considerably improved the efficiency of single-drug 5-FU therapy (Labianca *et al*, 1997). In addition, the discovery of several major active drugs, namely irinotecan and oxaliplatin, has completely modified our perception of chemosensitivity of colorectal cancer (Douillard *et al*, 2000; de Gramont *et al*, 2000). Palliative treatment of metastatic colorectal cancer is now based on the combination of folinic acid-modulated 5-FU (or another thymidylate synthase inhibitor) with either irinotecan or oxaliplatin, and allows to obtain response rates in the range of 40–60% with an overall survival benefit of more than 24 months (Taieb *et al*, 2005). This success has encouraged the use of such combinations in the adjuvant setting. Finally, the introduction of therapeutic antibodies directed against the EGF receptor or VEGF has further refined the treatment protocols (Cunningham *et al*, 2004; Moses *et al*, 2004).

The choice of the chemotherapy regimen appears critical: in view of the impressive survival advantage that can be expected in the palliative setting, and in view of the possible curability, it is crucial to offer to every patient the maximum likelihood of drug efficacy. We have currently no rational way to choose one combination over the other(s), in particular no predictive test allowing to select irinotecan or oxaliplatin to be combined with 5-FU. If there is some drug selectivity, it would be of utmost interest to identify the parameters that determine this selectivity to prescribe the drug or drug combination the most likely to provide a response and a survival advantage to a given patient. In addition, it would be also important to predict for drug-associated toxicity to avoid the prescription of a drug with a high risk of neutropoenia, diarrhoea or mucosal damage. Furthermore, we need to identify individual parameters that are predictive of drug-induced toxicity.

The pharmacology of irinotecan (Figure 1) is rather complex (Rivory and Robert, 1995) for several reasons: (i) it is a prodrug that needs activation to a metabolite, SN-38, to interact with its target, DNA-topoisomerase I (Top1); this activation, which is catalysed by carboxylesterase 2 (CES2), is believed to mainly occur in the liver, but *in situ* activation in the tumour cell cannot be ruled out; (ii) irinotecan and SN-38 are subject to metabolism to inactive species: irinotecan is detoxified by cytochrome P450 CYP3A4 to APC and NPC, whereas SN-38 is conjugated to glucuronic acid by UDP-glucuronosyl transferase UGT1A1 and possibly other isoforms; (iii) all camptothecins derivatives and metabolites are subject to spontaneous interconversion between a lactone (active) form and a carboxylate (inactive) form, depending on the pH of the fluid. Several enzymes involved in irinotecan metabolism present individual variations that may be of genetic origin: it is well known that the CYP3A4 liver concentrations may vary in a 1 : 20 ratio, which has been shown to depend on environmental factors rather than on gene polymorphisms (Lamba *et al*, 2002); furthermore, *UGT1A1* promoter is subject to a frequent polymorphism, leading to decreased activity, low SN-38 detoxification and increased risk for irinotecan toxicity (Iyer *et al*, 2002); finally, little is known about the polymorphisms of *CES2* but a polymorphism occurring in the promoter might well be responsible for decreased enzyme expression (Charasson *et al*, 2004).

We wanted in this study to establish the relationships that may exist between the genetic (gene polymorphisms) and non-genetic (CYP3A4 status) factors of variation of the metabolic transformations of irinotecan and the pharmacokinetics of the drug and its metabolites; and, further, to establish relationships between the pharmacokinetic or metabolic parameters and the efficacy and toxicity of irinotecan. A number of biological parameters potentially indicative of the drug elimination ability of the patients (plasma creatinine, bilirubin and liver enzymes) were studied at the onset of the treatment for studying their association with the pharmacokinetic parameters. This was achieved in a group of 49 patients treated for metastatic colorectal cancer with a combination of 5-FU and irinotecan; the pharmacokinetics of irinotecan and its metabolites were studied in all patients; the polymorphisms of UGT1A1 and CES2 were studied as potential markers for SN-38 glucuronidation and irinotecan activation, respectively; and the potential activity of CYP3A4 was estimated from cortisol biotransformation into 6*β*-hydroxycortisol (Yamamoto *et al*, 2000).

## Patients and methods

### Patients

The patients included in this study suffered from advanced or metastatic colorectal cancer, which had been histologically proven, and had never been treated with irinotecan. They could have received folinic acid-modulated 5-FU treatment as adjuvant or palliative therapy. Patients had to be between 18 and 85 years, with normal biochemical and haematological tests and a performance status ⩽2, evaluated as defined by the World Health Organization (WHO). They were followed regularly all along the evolution of the disease, and computed tomography scans were performed every 2 months for the evaluation of drug response and event-free survival. They were treated with the FOLFIRI regimen (André *et al*, 1999), and their treatment included folinic acid 400 mg m^−2^ as a 2-h i.v. infusion; 5-FU 400 mg m^−2^ i.v. bolus at J1, then 2400 mg m^−2^ as i.v. infusion over 46 h; and irinotecan 180 mg m^−2^ as a 1.5-h infusion. Courses were repeated every 2 weeks. Tumour response was assessed according to Response Evaluation Criteria in Solid Tumors (RECIST) criteria and in reviewing computed tomography scans. All adverse events, especially gastrointestinal events and leukopoenia were recorded and graded for severity according to WHO scales. All the patients enrolled had given informed consent to the study, which had been approved by the *Comité de protection des personnes dans la recherche biomédicale* of Bordeaux.

### Pharmacokinetic studies

For pharmacokinetic studies, blood samples were obtained from the first 28 patients, at the first course of treatment, at 1 and 1.5 h after the beginning of irinotecan infusion and then at 10, 30, 45 min, 1, 2, 4, 8, 12, 24 and 48 h after the end of irinotecan infusion. For the other patients, a limited sampling strategy was adopted and only four samples were obtained, namely at the end, and 10 min, 4 and 24 h after the end of irinotecan infusion. For the identification of gene polymorphisms, a blood sample was obtained at patient's inclusion, and leukocytes were prepared by Ficoll gradient centrifugation. For the evaluation of the CYP3A4 status, patients received, at least 24 h before chemotherapy, a dose of 300 mg of cortisol as an i.v. bolus. Urines were collected over 24 h by fractions of 3, 3 and 18 h, and blood samples were obtained 15 and 90 min after cortisol administration.

Irinotecan and metabolites were evaluated in plasma using an HPLC technique with fluorometric detection, which derives from a technique we developed earlier (Rivory and Robert, 1994). This technique allowed the separation and quantification of irinotecan and all its known metabolites, namely SN-38, SN-38G, APC and NPC. Briefly, 500 *μ*l of methanol/1 N hydrochloric acid (98 : 2, v/v) were added to 250 *μ*l of plasma. The tubes were vortex-mixed for 10 s, centrifuged at 10 000 **g** for 5 min at 4°C and 5 *μ*l of 1 N hydrochloric acid was added to 600 *μ*l of the supernatant. Later, after centrifugation at 10 000 **g** for 5 min at 4°C, 80 *μ*l of the supernatant was injected into the HPLC system. For SN-38G estimation, the samples were incubated with 1000 IU of *β*-glucuronidase for 2 h at 37°C prior to deproteinisation. The HPLC equipment consists of a Perkin Elmer 200 model with a fluorescence detector (FP-1520, JASCO, Bouguenais, France). Separation of compounds was achieved using a Symmetry Shield RP8 (5 *μ*m, 150 × 4.6 mm; Waters, Saint-Quentin-en-Yvelines, France) analytical column protected by a Symmetry Shield RP8 precolumn (5 *μ*m, 3.9 × 20 mm; Waters). The mobile phase A was performed with a mixture of 75 mM ammonium acetate buffer (adjusted to pH 6 with acetic acid)/acetonitrile (85 : 15, v/v). The mobile phase B was acetonitrile. A linear gradient from 0 to 25% phase B in 30 min was used at a rate of 1 ml min^−1^. The fluorescence detector excitation and emission wavelengths were set at 355 and 515 nm, respectively. The limit of quantification was 1 *μ*g l^−1^ for all products.

A limited sampling strategy was adopted for the 20 last patients included in the study, based on the study of Canal *et al* (1996). Individual pharmacokinetic parameters were calculated according to a nonlinear mixed effects approach using NonMem program (Version VI, level I). Irinotecan concentrations were adequately fitted with a three-compartmental model. Posterior Bayesian pharmacokinetic parameters were determined for each patient, especially clearance, which allowed to calculate irinotecan AUC. SN-38 plasma concentrations were analysed by considering irinotecan data and using two additional compartments (i.e., central and peripheral compartment for SN-38). SN-38G and APC concentrations were analysed separately. For both SN-38G and APC, a two-compartment model with first-order input was used. For the validation of the limited sampling strategy, the AUC values of irinotecan and its metabolites were calculated for the 28 data-rich patients using the trapezoidal rule (non-compartmental approach) and compared with those obtained using the population approach.

### Metabolic and pharmacogenetic studies

Cortisol and 6*β*-hydroxycortisol were evaluated in urine using an HPLC technique (Rouits *et al*, 2003). Extraction was performed using an ethyl acetate/isopropanol mixture (85 : 15, v/v). HPLC was performed, as described earlier, using UV absorbance at 244 nm for detection.

Genomic DNA was extracted from leukocytes using the QIAamp® DNA minikit from Qiagen or the DNA Isolation Kit from Roche Molecular Diagnostics (Meylan, France). It was quantified by spectrophotometry. Polymerase chain reactions (PCR) were performed on genomic DNA using appropriate primers. Two polymorphisms were sought in genomic DNA: the TA repeat in the *UGT1A1* gene promoter (UGT1A1^*^28 genotype, rs8175347) and an SNP in the promoter of the CES2 gene (830C>G; rs11075646). UGT1A1^*^28 genotype was determined using the technique of Pyrosequencing (Rouits *et al*, 2004). The *CES2* genotype was determined by sequencing of the amplified PCR products obtained using the following primers: forward: 5′-CTCCTGGGGTCTCCAATTCT-3′; reverse: 5′-GAAAGGTGGGTGTGGTAGGA-3′.

### Statistical studies

Statistics were performed using SPSS software (Chicago, IL, USA). The *α*-error risk was classically chosen as 5%. All statistical tests were bi-directional. Continuous variables were compared using the Pearson coefficient of correlation. Fisher's exact test was used to study the relationships between drug response and toxicity and the parameters studied. Owing to the high number of tests performed, the Bonferroni correction was applied to decrease the probability of detecting falsely positive relationships.

## Results

### Clinical outcome

A total of 49 colorectal cancer patients entered the study; there were 34 males and 15 females and their median age was 60 years, similar for both genders (Table 1). All of them were treated according to the protocol described above, with a total of 190 courses of treatment. Toxicity and overall survival were evaluable for all patients, response for 47 patients and progression-free survival for 35 patients (Table 1). Overall survival was 20% at 2 years. The median follow up of the patients was 540 days from the onset of chemotherapy.

### Pharmacokinetics of irinotecan

Pharmacokinetic studies were performed during the first course of treatment in 28 patients with 10–13 blood samplings and in 20 additional patients with a limited sampling strategy. The UGT1A1 polymorphism was determined in 44 patients, the CES2 polymorphism in 48 patients and the 6*β*-hydroxycortisol/cortisol urinary ratio (measured 3, 6 and 24 h after the administration of 300 mg cortisol) in 46 patients.

The mean pharmacokinetic parameters of irinotecan and its metabolites as obtained using the Bayesian approach are presented in Table 2. Comparison of AUC values obtained using the trapezoidal rule and the population approach was done for the 28 patients with data-rich samplings and revealed coefficients of correlation of 0.96, 0.95, 0.93 and 0.99 for irinotecan, SN-38, SN-38G and APC, respectively. Bayesian AUC values were used for further correlation analyses.

There was a weakly significant correlation between the dose administered and the AUC values of irinotecan and SN-38 (*r*=0.311 and 0.265, respectively, *P*=0.03 and 0.07). There were also significant correlations between the AUC of irinotecan and those of each of the metabolites (0.309<*r*<0.522, 0.0002 <*P*<0.03). Only a slight, nonsignificant correlation was found between the AUC values of SN-38 and SN-38G (*r*=0.211, *P*<0.15). A highly significant correlation existed between the AUC values of APC and NPC (*r*=0.857, *P*<10^−7^). It is also remarkable that the relative extent of glucuronidation of SN-38 (as defined by the ratio of the AUC of SN-38G to that of SN-38, see Rivory *et al*, 1997) was inversely correlated with the relative extent of the activation of irinotecan (as defined by the ratio of the AUC of SN-38 to that of irinotecan) (*r*=–0.426, *P*=0.002).

### Relationships between clinicobiological and pharmacokinetic parameters

**Figure 2:**
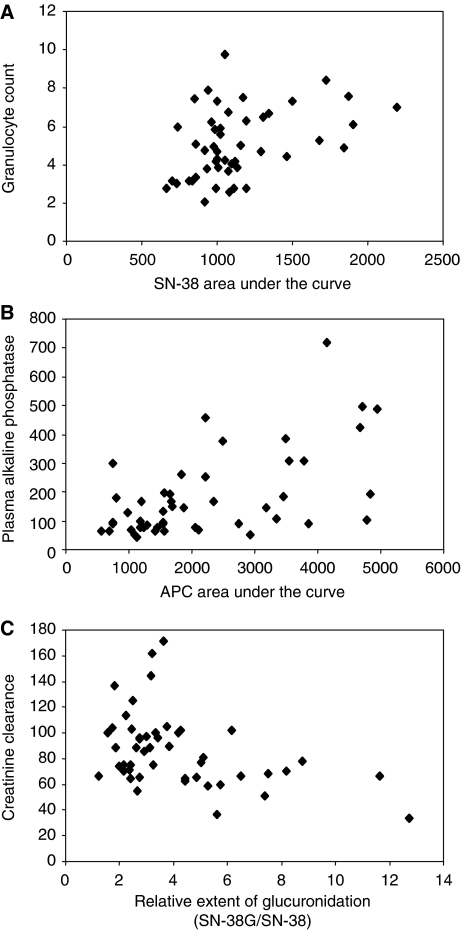
Charts representing the correlations between (**A**) SN-38 AUC value (mg h^−1^ l^−1^) and pretreatment granulocyte counts ( × 10^9^) *r*=0.402, *P*=0.005; (**B**) APC AUC value (mg h^−1^ l^−1^) and pretreatment plasma alkaline phosphatase (IU l^−1^) *r*=0.576, *P*=2 × 10^−5^; (**C**) Relative extent of glucuronidation (SN-38G/SN-38) and pretreatment creatinine clearance (ml min^−1^) *r*=0.412, *P*=0.004.

No relationship between treatment response, progression-free survival or toxicity and any of the pharmacokinetic parameters was evidenced. In contrast, several important biological constants were significantly correlated to pharmacokinetic parameters. Limited to the significant relationships after Bonferroni correction, we observed that the pretreatment leukocyte and granulocyte counts were significantly correlated with SN-38 AUC (or the relative extent of activation) (*r*=0.402, *P*=0.004, Figure 2A), that a series of hepatic parameters (alkaline phosphatase, transaminases and lactate dehydrogenase) were significantly correlated with the AUC values of APC (or the relative extent of metabolisation) (0.456<*r*<0.576, 2 × 10^−5^<*P*<0.0001, Figure 2B) and that plasma creatinine was significantly correlated (or creatinine clearance inversely correlated) with the AUC value of SN-38G (or the relative extent of glucuronidation) (0.318<*r*<0.412, 0.004<*P*<0.03, Figure 2C).

### Relationships between pharmacokinetic parameters and metabolic predictors

The rare allele (seven TA repeats) frequency of *UGT1A1* was 0.30 and the rare allele frequency (G) of *CES2* was 0.14. Both genotype distributions followed the Hardy–Weinberg equilibrium. There was no relationship between the polymorphisms of *UGT1A1* or *CES2* and any of the pharmacokinetic parameters obtained, the mean values of each parameter being not significantly different in wild-type or variant patients for either polymorphism. The relationship between *UGT1A1* polymorphism and SN-38G AUC value especially failed to reach significance (*P*=0.20). In contrast, there was a consistent association between the 6*β*-hydroxycortisol/cortisol ratio at 6 h after cortisol injection and the AUC values of APC (or the relative extent of metabolisation) (*r*=0.437, *P*=0.002).

### Relationships between clinicobiological parameters and metabolic predictors

The relationships between the three metabolic predictors (*UGT1A1* and *CES2* polymorphisms and the 6*β*-hydroxycortisol/cortisol ratio) and the clinical and biological parameters could be studied in the whole set of patients, independent of the pharmacokinetic data. The *UGT1A1* polymorphism was significantly related to the bilirubin plasma level: variant homozygous patients had a mean bilirubin plasma level of 2.5 higher than heterozygous (*P*=3 × 10^−6^) or common homozygous patients (*P*=7 × 10^−7^) (Table 3). This was also the case for ALT transaminase when comparing common and variant homozygous patients (*P*=0.004). Variant homozygous patients also had significantly lower plasma sodium levels than heterozygous or common homozygous patients, but the difference was only 3.6% (*P*=0.001). This polymorphism was a significant predictor of toxicity: 3 patients out of 23 common homozygous underwent an episode of grade >1 neutropoenia during at least one of the four first courses of treatment, whereas this was the case for 9 patients out of 21 heterozygous or variant homozygous (*P*=0.042 with Fisher's exact test). No relationship could be evidenced between *UGT1A1* polymorphism and treatment response- or progression-free survival (Table 3).

The *CES2* polymorphism was unrelated to any of the biological parameters analysed, with the exception of plasma lactate dehydrogenase, which was twice lower in common homozygous patients than in patients having at least one variant allele (*P*=0.02, not significant after the Bonferroni correction). This polymorphism was unrelated to treatment response or progression-free survival, but appeared as a modest predictor of diarrhoea: 8 patients out of 36 common homozygous underwent an episode of grade >1 diarrhoea during at least one of the four first courses of treatment, whereas this was the case for 1 patient out of 12 patients having at least one variant allele (not significant) (Table 3).

The urinary 6*β*-hydroxycortisol/cortisol ratio was studied at three different time intervals after the administration of 300 mg cortisol. There was a significant positive correlation between the 6-h ratio and the plasma transaminases level (*r*=0.501, *P*=0.0003). There was a significant association between the 6-h (and the 24-h) ratio and the risk of grade >1 toxicity: patients who had encountered such an episode of neutropoenia or diarrhoea during any of the four first courses of treatment had a 1.84-fold higher 6-h ratio than patients who had not (*P*=0.022). From these data, it was possible to define a threshold above which the risk of toxicity is maximal; using the method of Youden, it was possible to optimise this threshold at 0.52, giving a sensitivity of 80% and a specificity of 77%. The association between a high 6-h ratio and the risk of toxicity is especially marked for diarrhoea: only 2 patients out of 31 with a ratio <0.52 underwent grade >1 diarrhoea, whereas this was the case for 8 patients out of 15 with a ratio >0.52 (*P*=0.003, with the Fisher exact test) (Table 3).

## Discussion

The mean pharmacokinetic and metabolic parameters we obtained for the 49 patients by Bayesian analysis were within the range of the values usually obtained (Mathijssen *et al*, 2003) and the dispersion of the values is also comparable. The correlation between the dose of irinotecan administered and the AUCs of both irinotecan and SN-38 has been observed in most pharmacokinetic studies. The correlation between APC and NPC AUCs was expected in view of the fact that these two metabolites are produced by the same enzyme, CYP3A4, in two parallel pathways (Haaz *et al*, 1998a, 1998b). However, the inverse correlation between the relative extent of SN-38 glucuronidation and the relative extent of irinotecan activation has never been mentioned in the published pharmacokinetic studies. In other words, a high glucuronidation rate is associated with a low rate of formation of SN-38. This would tend to indicate that the formation of the glucuronide is independent of the availability of SN-38 and that a fixed amount of SN-38 is transformed into SN-38G, suggesting a saturation process.

There was no direct relationship between the pharmacokinetic parameters and the clinical outcome, both for toxicity and efficacy. Such pharmacokinetic–pharmacodynamic relationships have been found in some studies (de Forni *et al*, 1994; Abigerges *et al*, 1995; Sasaki *et al*, 1995; Canal *et al*, 1996) but not all (Catimel *et al*, 1995; Rothenberg *et al*, 1996; Herben *et al*, 1999; Ma *et al*, 2000). For instance, neutropoenia and diarrhoea could be predicted from irinotecan and SN-38 AUCs in three original studies (Abigerges *et al*, 1995; Sasaki *et al*, 1995; Canal *et al*, 1996). However, such correlations were obtained in early dose-finding studies with a large range of doses prescribed and, therefore, a large range of AUC values. In phase III studies performed at a fixed dose, the AUC range is usually too small to identify pharmacokinetic–pharmacodynamic relationships.

A careful analysis of the relationships between the pharmacokinetic parameters and the pretreatment biological constants has revealed several important features. The leukocyte count was associated with SN-38 AUC, which would suggest that a significant part of the plasma SN-38 might originate from blood-nucleated cells. The role of leukocytes in the activation of irinotecan has been mentioned in a study of Cecchin *et al* (2005) who observed an association between *CES2* mRNA expression in peripheral blood mononuclear cells and the level of irinotecan activation in patients' plasma. The correlation between the level of conversion of cortisol into 6*β*-hydroxycortisol, and the plasma levels of APC is in agreement with the major role of CYP3A4 in this pathway of transformation of irinotecan (Haaz *et al*, 1998b). The correlation between plasma liver enzymes and the AUC of APC was unexpected, as it has been shown that CYP3A activity was reduced by 50% in patients with concurrent elevations in liver transaminases (Baker *et al*, 2004); however, it has also been shown that CYP3A4 expression was associated with the occurrence of metastases in osteosarcoma (Dhaini *et al*, 2003), which might explain why patients with high cortisol conversion into 6*β*-hydroxycortisol had altered liver enzymes. Another interesting observation is that creatinine clearance was inversely correlated with SN-38G AUC. This would indicate that renal function plays a major role in the disposition of SN-38G and that the elimination of this metabolite is a determinant of its plasma concentration as important as its formation from SN-38.

The UGT1A1^*^28 polymorphism did not appear to be significantly related to the SN38-G AUC value or to the extent of glucuronidation as has been observed in other studies (Iyer *et al*, 2002; Rouits *et al*, 2004; Toffoli *et al*, 2006; Côté *et al*, 2007; Ramchandani *et al*, 2007) whereas, as expected, the bilirubin plasma levels were much higher in the variant homozygous patients than in common homozygous and heterozygous patients. Despite the absence of significant association with the glucuronidation rate of SN-38, the *UGT1A1* polymorphism appeared to be significantly associated with irinotecan toxicity, the patients having at least one variant allele being at increased risk of neutropoenia. This has been already shown to be in relation with the decreased transcription rate of *UGT1A1* when the promoter harbours seven TA repeats, and to the subsequent decrease of enzyme dosage for SN-38 detoxification. Our study thus confirms the importance of the pre-therapeutic determination of the *UGT1A1* genotype for predicting irinotecan toxicity, and indicates that heterozygous patients may be also at increased risk for neutropoenia. Bilirubinemia by itself did not appear as a predicting factor for irinotecan toxicity.

It is remarkable that the CYP3A4 status, defined by the 6*β*-hydoxycortisol/cortisol ratio, appears as a predictor of the diarrhoea and, to a lesser extent, to the neutropoenia undergone by the patients, which is an original and important observation. Few studies, until recently, have evaluated the pharmacokinetics of these metabolites, and no study establishing metabolic–pharmacodynamic relationship is presently available. The mechanism by which the CYP3A4 status influences the occurrence of irinotecan-induced diarrhoea remains elusive, as the metabolites of irinotecan produced in this pathway do not display cytotoxic activity. It might be simply related to the hepatic dysfunction that is associated with high cortisol biotransformation in this group of patients. Nevertheless, it appears tempting to propose the use of a pretreatment CY3A4 determination for predicting this major side effect of irinotecan, using, for instance, the cortisol 6*β*-hydroxylation approach or another one, such as the determination of midazolam clearance, which has been shown to be significantly associated with irinotecan clearance (Mathijssen *et al*, 2004).

As a conclusion, our studies have revealed several interesting tracks concerning the prediction of irinotecan toxicity and some new insights on the metabolism–pharmacodynamic relationships of this major drug. We confirm the use of *UGT1A1* genotyping to predict for haematological toxicity, both for homozygous and heterozygous patients; we demonstrate also the potential importance of CYP3A4 status to predict for the occurrence of digestive toxicity. We show in addition that SN-38G plasma levels are associated with renal function, that the participation of blood cells in irinotecan activation might be more important than previously thought and that the extent of glucuronidation of SN-38 is inversely related to the relative formation of SN-38 from irinotecan. All these observations may improve the routine use of irinotecan in colorectal cancer patients.

## Figures and Tables

**Figure 1:**
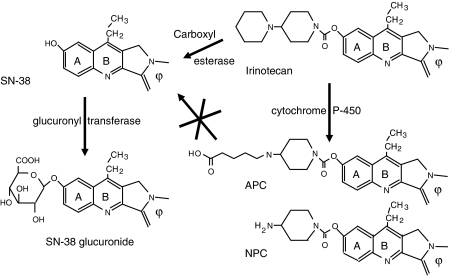
The metabolism of irinotecan in humans.

**Table 1:** Clinical features of the patients entering the study

Patients included	49
Gender (M/F)	34/15
Median age (range)	60 (33–78)
Performance status (0/>0)	27/20
Tumour site (colon/rectum/both)	29/16/4
Metastases (hepatic/extrahepatic)	35/14
Prior adjuvant therapy (yes/no)	19/30
Prior palliative therapy (yes/no)	9/40
Overall response (progression/stable disease/objective response)	10/25/11
Neutropoenia over 190 courses (grade 1/grade 2/grade⩾3)	20/5/9
Diarrhoea over 190 courses (grade 1/grade 2/grade 3)	38/10/3
Median progression-free survival (days)	92
Median overall survival (months)	13

**Table 2:** Pharmacokinetic parameters of irinotecan and metabolites in 48 patients (Bayesian population approach)

*Pharmacokinetic parameters of irinotecan*	
Total plasma clearance	31.5±6.8 l h^−1^
Volume of distribution at steady state (*V*_ss_)	328±253 l
	
*AUCs of irinotecan and metabolites (mg h l* ^*−1*^ *)*	
Irinotecan	10 530±2713
SN-38	1133±337
SN-38G	4369±2165
APC	2205±1293

Results are given as mean±s.d.

**Table 3:** Relationships between the metabolic predictors and efficacy and toxicity of the treatment

**Predictor**	**Genotype (patient nos.)**	**Neutropoenia**	**Diarrhoea**	**Response**	**Bilirubin**	**ALT**
UGT1A1	WT (23)	3 (13%)	4 (17%)	3/13/7	8.5±3.1	24.2
		(0.04–0.23)	(0.06–0.38)			
	HT (16)	9 (43%)^*^	4 (19%)	6/4/5	7.5±3.3	31.1
	VAR (5)	(0.24–0.63)	(0.07–0.40)	0/4/1	18.6±3.5^**^	63.0^***^
						
CES2	WT (36)	8 (22%)	8 (22%)	7/15/12	9.6±4.6	33.7
		(0.12–0.38)	(0.12–0.38)			
	HT/VAR (11/1)	4 (33%)	1 (8%)	3/7/2	8.5±4.5	23.0
		(0.14–0.61)	(0–0.38)			
						
Ratio 6*β*-hydroxycortisol/cortisol						
	<0.52 (31)	5 (23%)	2 (6%)	7/17/7	8.9±3.9	26.4
		(0.07–0.33)	(0–0.22)			
	>0.52 (15)	7 (47%)	7 (47%)^****^	3/6/7	10.0±5.6	40.2
		(0.25–0.70)	(0.25–0.70)			

For UGT1A1 and CES2, the genotypes were homozygous wild type (WT), heterozygous (HT) or homozygous variant (VAR). For the CYP3A4 status, the ratio 6*β*-hydoxycortisol/cortisol was split at a threshold of 0.52, as described in the article. The number of patients in each group is indicated in text. For toxicity data, the number of patients undergoing grade >2 toxicity (WHO grading) is indicated, with the corresponding percentage between parentheses and the 95% confidence intervals between parentheses. For response, the number of patients with progression/stabilisation/objective response is indicated, using RECIST criteria. Mean bilirubin (*μ*mol l^−^) and ALT values (UI l^−1^) for each group of patients are indicated.

Asterisks indicate a significant difference between patients' groups: ^*^P=0.042; ^**^P=3 × 10^−6^; ^***^P=0.004; and ^****^P=0.03.
